# Inter-application displacement of brachytherapy dose received by the bladder and rectum of the patients with inoperable cervical cancer

**DOI:** 10.2478/raon-2013-0082

**Published:** 2014-04-25

**Authors:** Goran Marosevic, Dzenita Ljuca, Hasan Osmic, Semir Fazlic, Oliver Arsovski, Dusan Mileusnic

**Affiliations:** 1 Centre for Radiotherapy, International Medical Centre Banja Luka, Banja Luka, Bosnia and Herzegovina; 2 Gynaecology and Obstetrics Clinic, University Clinical Centre Tuzla, Tuzla, Bosnia and Herzegovina; 3 Department for Radiotherapy, University Clinical Centre Tuzla, Tuzla, Bosnia and Herzegovina

**Keywords:** inter-application variations, brachytherapy, inoperable cervical cancer

## Abstract

**Background:**

The aim of the study was to examine on the CT basis the inter-application displacement of the positions D_0.1cc_, D_1cc_ and D_2cc_ of the brachytherapy dose applied to the bladder and rectum of the patients with inoperable cervical cancer.

**Patients and methods:**

This prospective study included 30 patients with cervical cancer who were treated by concomitant chemo-radiotherapy. HDR intracavitary brachytherapy was made by the applicators type Fletcher tandem and ovoids. For each brachytherapy application the position D_0.1cc_ was determined of the bladder and rectum that receive a brachytherapty dose. Then, based on the X, Y, and Z axis displacement, inter-application mean X, Y, and Z axis displacements were calculated as well as their displacement vectors (R). It has been analyzed whether there is statistically significant difference in inter-application displacement of the position of the brachytherapy dose D_0.1cc_, D_1cc_ and D_2cc_ of the bladder and rectum. The ANOVA test and post-hoc analysis by Tukey method were used for testing statistical importance of differences among the groups analyzed. The difference among the groups analyzed was considered significant if p < 0.05.

**Results:**

There are significant inter-application displacements of the position of the brachytherapy dose D_0,1cc_, D_1cc_ and D_2cc_ of the bladder and rectum.

**Conclusions:**

When we calculate the cumulative brachytherapy dose by summing up D_0,1cc_, D_1cc_ and D_2cc_ of the organs at risk for all the applications, we must bear in mind their inter-application displacement, and the fact that it is less likely that the worst scenario would indeed happen.

## Introduction

Intracavitary brachytherapy has for decades been an obligatory type of the treatment of the locally advanced cervical cancer. The basic principles of brachytherapy are based in the traditional schools (Paris, Manchester, Stockholm, Fletcher, etc.); any are still dominant in planning the brachytherapy for the cervical cancer.^1^,^2^ Verification of the applicator position, as well as of the organs at risk (the bladder and rectum, as well as the sigmoid for 3D brachytherapy) is done with the aim of optimizing the brachytherapy dose in order to achieve a complete distribution of the dose around the target volume, with maximum sparing of the organs at risk. The prescribing of the dose is made by the standard Manchester system of the dose to the point A.^3^ Nowadays, especially in the developed countries, CT (computer tomography) and MR (magnetic resonance) based brachytherapy is becoming standard in treating gynaecological tumours, particularly those locally advanced.^4^–^8^ 3D MRI could potentially replace multiplanar 2D MRI in cervix cancer IGABT (image guided adaptive MRI based brachytherapy), shortening the overall MRI scanning time and facilitating the contouring process, thus making this treatment method more widely employed.^9^ Intracavitary brachytherapy of cervical cancer consists of multiple applications, usually four to five. As recommended by the GECESTRO work group, it is important for the 3D image guided adaptive MRI based brachytherapy of cervical cancer to verify what is the minimum dose received by the most irradiated 0.1 cm^3^, 1 cm^3^ and 2 cm^3^ (D_0.1cc_, D_1cc_ and D_2cc_, respectively) of the bladder and rectum volume.^10^ The doses received by organs at risk for all brachytherapy applications are summed up together with the external dose, and by using the linear-quadratic model, the total cumulative dose is determined.^11^ In planning brachytherapy CT does not give us the possibility to precisely delineate tumour and plan the distribution of the therapy dose to the tumour (as is the case with MR planning). However, it is possible to obtain precise data on the contribution of the brachytherapy dose to the organs at risk.^12^,^13^ Georg *et al.*, correlated the level of complications with the dose received by the above mentioned referential volumes of the organs at risk.^14^ Recently, Hollowey *et al.* published the results including the application related variation of the dose received by the sigmoid, under the conditions that the same volume of sigma always receives the brachytherapy dose.^15^ However, provided that there are inter-application displacements of D_0.1cc_, D_1cc_ and D_2cc_ of the organs at risk, the question is how much of and which volume of the organs at risk receives the calculated cumulative dose for all the fractions? Does the worst case scenario exist at all?

The aim of our study was to examine on the CT basis the inter-application displacement of the positions D_0.1cc_, D_1cc_ and D_2cc_ of the brachytherapy dose applied to the bladder and rectum of the patients with inoperable cervical cancer.

## Patients and methods

This prospective study included the patients with cervical cancer FIGO IIb-IVa stage, who were treated by concomitant chemo-radiotherapy at the University Clinical Centre Tuzla, at the Department for Radiotherapy of the Clinic for Oncology, Haematology and Radiotherapy. The study was conducted on a consecutive sample of 30 patients treated in the period April 2010 – May 2012. The inclusion criteria were non-operated patients; brachytherapy was made with an intra-uterus applicator and two vaginal ovoids. The investigators followed recommendations of the Helsinki Declaration. The study protocol was approved by the ethic committees of the University Clinical Centre Tuzla.

The patients were treated by the external radiotherapy to the pelvis by the tumour dose (TD) 45 Gy in 25 fractions along with the concomitant chemotherapy with cisplatin with the dose of 40 mg/m^2^. External radiotherapy was applied by the linear accelerator ElektaSinergy^®^ and the energy of 15 MV. After 10 to 13 fractions of the external radiotherapy, intracavitary brachytherapy was started. The intracavitary brachytherapy was applied by the applicators type Fletcher tandem and ovoids, once a week at the high dose rate (HDR) regime with Iridium (^192^Ir) on Flexitron^®^. Protocols for the rectum and bladder filling required that the patients took 20 mg bisacodyl laxative suppositories (Dulcolax^®^) 12 hours prior to every brachytherapy application and that they urinated immediately before every brachytherapy application. During every application, a tamponade towards the urinary bladder and rectum was made by the gauze soaked in the lopromide (Ultravist^®^) contrast liquid which was in 4 to 1 ratio with the physiological solution. The therapy dose of (TD 7 Gy) was determined in accordance to the Manchester system to the A point.

After each brachytherapy application (five in total), computer tomography of the pelvis was made. During every CT scan, on the previously marked referential spots needed for the external radiotherapy, 3-mm diameter small lead balls were fixated, and the patients were positioned in such a way that the referential marks corresponded to the laser coordinate system of the CT scanner. This way, patient’s geometry was connected to the geometry of the CT scanner, fulfilling the condition that during each computer tomography scan the patient is in the same position.

After every computer tomography the delineation of organs at risk (the bladder and rectum) was made. The bladder and rectum delineation was made on every CT slice: for the rectum at 1 cm from the anus to the recto-sigmoid transition through the entire thickness of the organ wall, and for the bladder following the outer contour of the entire organ volume. The planning of brachytherapy dose distribution for each application was made on the basis of computer tomography with the software system for planning Flexiplan Isodose Control^®^.

For each application D_0.1cc_, D_1cc_ and D_2cc_ for the bladder and rectum were calculated. The abovementioned planning system was used for the formation of co-ordinate system whose axes X (lateral), Y (antero-posterior), and Z (cranial-caudal) were lying on the referential marks (small lead balls), since they have a constant value and represent the pelvis as one co-ordinate system. In this co-ordinate system, for each application the position D_0.1cc_ was determined for bladder and rectum. Considering the fact that D_0.1cc_ is located in D_1cc_ and D_2cc_, it therefore represents their position as well. Then, on the basis of X, Y, and Z axis displacements, the mean inter-application X, Y, and Z displacements were calculated, as well as their absolute displacements, that is displacement vectors (R). We analyzed whether there is a statistically significant difference in the inter-application displacement of the position of the brachytherapy dose D_0.1cc_, D_1cc_ and D_2cc_ for the bladder and rectum between the planning for all applications in relation to the first application. A post-hoc analysis was made of the position displacement from one application to another.

In the statistical processing of the results, standard methods of descriptive statistics have been used (arithmetic mean with the standard deviation and the numerical range from minimum to maximum value). For testing the statistical significance of differences among the examined groups ANOVA test was used as well as the post-hoc analysis by Tukey. Statistical hypotheses were tested at the significance level of *α* = 0.05, *i.e*. the difference p < 0.05 was considered statistically significant. SPSS 17.0 (SPSS Inc, Chicago, IL) statistics software was used for the data analysis.

## Results

Thirty patients were included in the study. A total of 150 brachytherapy applications were made. Table 1 shows the patient demographics. The average age of the patients at the time of the treatment was 52, a most of them were at FIGO IIb stage of planocelular cervical cancer.

**FIGURE 1.**
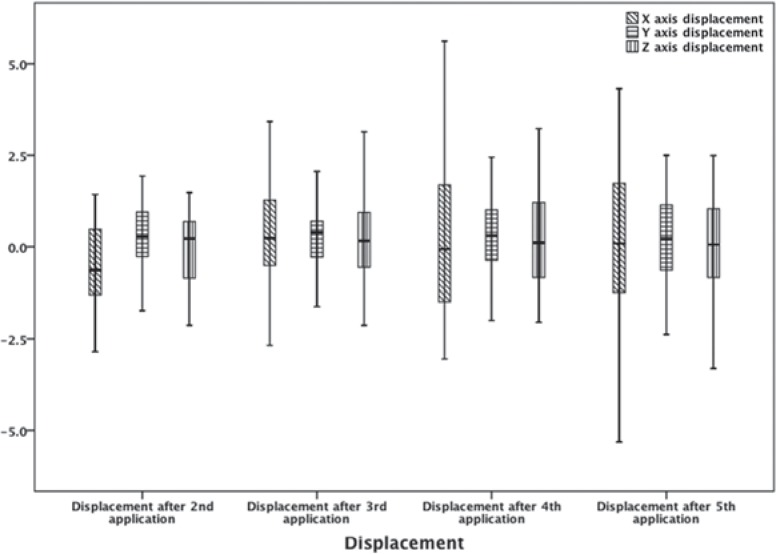
The mean values of the inter-application X, Y, and Z axis displacements of the brachytherapy dose on the bladder for each application in relation to the first one. Also, the figure shows the differences between every fraction individually. The results are given in centimetres (cm).

**FIGURE 2.**
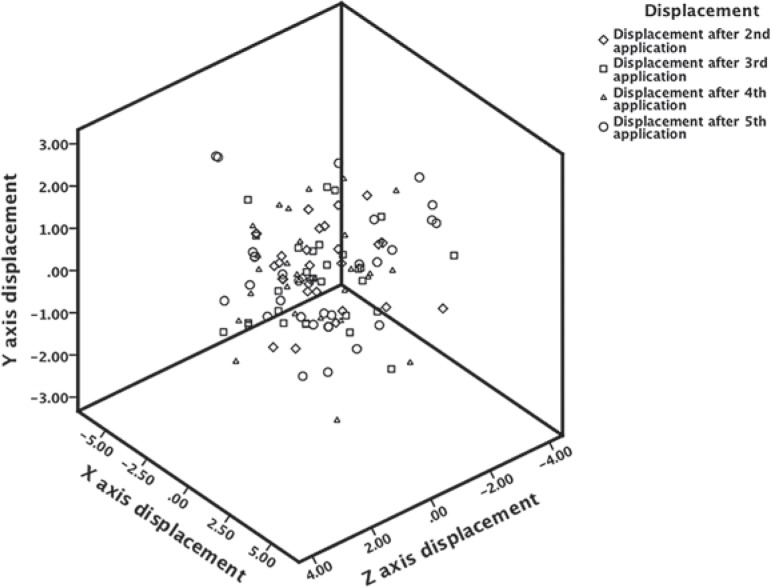
Absolute displacements, that is the vector of inter fraction displacements of the brachytherapy dose on the bladder given in centimetres for every fraction in relation to the first one, which is presented by the centre of the co-ordinate system.

The results of the mean values of inter-application X, Y, and Z axis displacements of the brachytherapy dose on the urinary bladder for each application in relation to the first one are given in Figure 1 and Table 2. The absolute displacements, that is, their displacement vector is represented in a three-dimensional figure (Figure 2). The range of vector magnitude of the brachytherapy dose received by the referential volumes of the bladder was 1.95 to 2.83 cm. The post-hoc analysis by Tukey shows for the absolute displacement that the significant difference by ANOVA analysis is due to a statistically significant difference among absolute displacements after applications II and V; p = 0.018 (Table 2).

**FIGURE 3.**
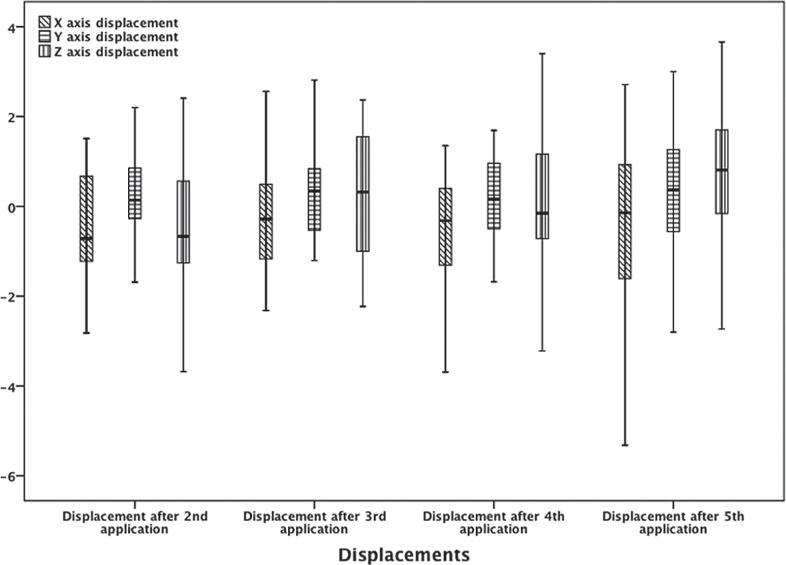
The mean values of the inter-application X, Y, and Z axis displacements of the brachytherapy dose on the rectum for each application in relation to the first one. Also, the figure shows the differences between every fraction individually. The results are given in centimetres (cm).

**FIGURE 4.**
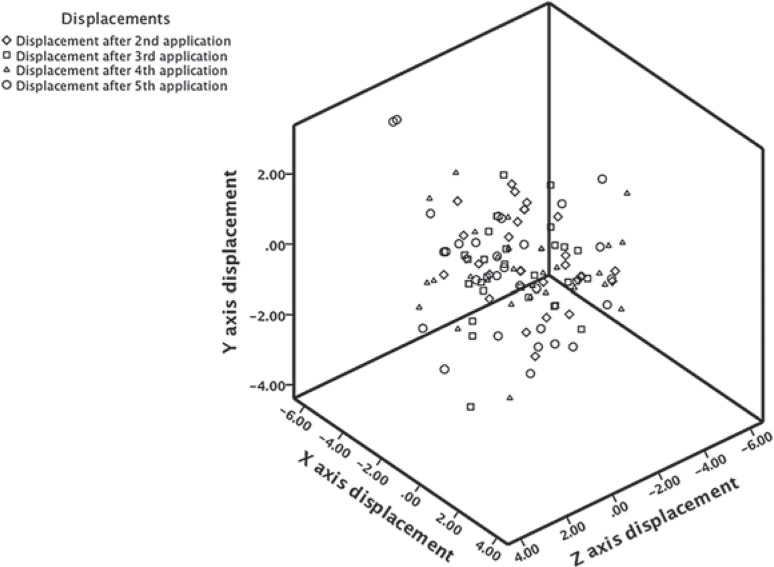
Absolute displacement that is the vector of interfraction displacements of the brachytherapy dose on the rectum given in centimetres for every fraction in relation to the first one, which is presented by the centre of the co-ordinate system.

The results of the mean values of the inter-application X, Y, and Z axis displacements of the brachytherapy dose to the rectum for each application in relation to the first one are given in Figure 3 and Table 3, while the absolute displacements, that are their displacement vector, are shown in a three-dimensional figure (Figure 4). The range of vector magnitude of the brachytherapy dose received by the referential volumes of the rectum was 2.05 to 2.78 cm. The post-hoc analysis by Tukey shows for the absolute displacement that the significant difference by ANOVA analysis is due to a statistically significant difference among absolute displacements after applications II and V; p = 0.038 and applications III and V; p = 0.023 (Table 3).

## Discussion

No publications currently available showing displacement of the brachytherapy dose received by the referential volumes of organs at risk from one application to another, for patients treated against inoperable cervical cancer. By analyzing the results of the average values of inter-application X, Y, and Z axis displacements of the brachytherapy dose on the bladder, which are shown in Figure 1 and Table 2, it can be noticed that the mean values of displacements are not of statistical significance. However, one should pay attention to minimum and maximum variations that are not irrelevant, especially on the X axis. Considering that the displacement of irregular three-dimensional volumes is analyzed in three-dimensional space, it is important to show the results of the absolute displacement, that is their vectors, in three-dimensional space.

Figure 2 shows a three-dimensional co-ordinate system which presents inter-application displacement vectors of the brachytherapy dose received by the referential volumes of the bladders. Besides the fact that it is evident that the vectors of the volumes analyzed do not overlap at a single point (and even if they did, they would probably not be-long to the same patient), this figure also shows the position of the referential volumes of the bladder in relation to the first application which represents the centre of the co-ordinate system, as well as the positions of volumes from one application to another. By observing the three-dimensional vectors of all the applications, the impression is that in the entire space they have a form of a ball. Statistically, a significant difference has been shown in inter-application displacements of the referential volumes of the bladder that receive the brachytherapy dose, and the difference between the second and fifth application is especially important (Table 2).

The displacements of the bladder during the transcutaneous radiotherapy have already been proved in the study by Ahmad R *et al.*, although the patients were in a prone position and immobilized by a belly board.^16^

The recommendations by the Gynaecological (GYN) GEC-ESTRO Working Group (IV) for MR imaging within the frame of image based adaptive cervix cancer brachytherapy suggest that prior to MR imaging a folley catheter is inserted, the urinary bladder is emptied, 50 ml of salt solution is injected, and then the procedure is repeated immediately before the very delivery of the brachytherapy dose.^17^ During the preparation of the patients for brachytherapy in this study, a folley catheter was not inserted into the urinary bladder. The planning was not made on MRI basis, and the urinary bladder wall was clearly visible, especially the back side wall, as the gauze soaked in the Ultravist^®^ contrast liquid clearly demarked the front vaginal fornix from the back wall of the bladder. The results of the mean values of inter-application X, Y, and Z axis displacements of the brachytherapy dose on the rectum, presented in Figure 3 and Table 3, show that there is no statistical significance. However, there are also extreme minimum and maximum values for all the axes, while the statistical significance for Z axis is at the level of P = 0.08. These extreme values of the displacements of the brachytherapy dose received by the referential volumes of the urinary bladder and the rectum which are obtained in this study are not that incomprehensible.

Namely, the measurements of the brachytherapy dose position are determined in relation to the centre of the co-ordinate system (whose axes lie on lead marks), which was positioned in the virtual centre of the pelvis, and not in relation to the brachytherapy applicator. This methodology was chosen with the aim to determine the real inter-application displacements of the brachytherapy dose, given by all the possible factors combined. These factors include: inter-application displacements of the applicator position, inter-application displacements of the cervix and tumour, different degree of tamponing the vaginal fornicis, physiological movements, and changes in the volume of the rectum and bladder.

Figure 4 shows a three-dimensional co-ordinate system for the rectum, in which inter-application displacements are visible of the vector of the maximum brachytherapy dose received by the referential volumes. Statistically significant difference exists here, especially between the second and fifth application and the third and fifth application (Table 3). It is difficult to explain with certainty why the inter-application displacements of the brachytherapy dose received by the referential volume of the rectum are more frequent than those of the bladder volume. The reason may lie in the fact that the volume of the rectum, as the organ which can potentially receive a maximum brachytherapy dose, is bigger. Also, the rectum has a higher possibility of drastically changing its volume due to gases. Haripotepornkul *et al.* analyzed both inter and intra-fraction displacements of the cervix during the IMRT, and, as one of the reasons for the cervix displacement they stated the gases, which at a certain point lead to a higher exposure of the rectum to the therapy dose.^18^ Other authors noticed this problem as well, but they also concluded that it is not easy to solve, as the application of laxatives is not efficient since it only decreases the solid matter in the rectum.^19^,^20^

Physiological movements of the intestines, that is, peristaltic, can be one of the factors which, during the brachytherapy treatment, lead to the displacement of the brachytherapy dose on the rectum wall. Physiological movements of the intestines can be reduced by the application of intravenous and intramuscular drugs, as in the preparation of the MRI based “image guided” adaptive brachytherapy of the cervical cancer.^16^ However, it has not been examined to what extent it affects the inter-application displacement of the brachytherapy dose on the front wall of the rectum.

The disadvantage of this study is that the displacement of D_0,1cc_ represented the displacement D_1cc_ and D_2cc_. However, D_1cc_, and especially D_2cc_ are extremely irregular volumes that change their shape from one application to another. Right now, the literature does not contain a published methodology that might analyze this problem in a more appropriate way in terms of three-dimensional view. Therefore, to analyze them as dotted structures (D_0,1cc_) is currently closest to the truth for this type of research.

## Conclusions

During the brachytherapy of the inoperable cervical cancer, there is a significant inter-application displacement of the positionsD_0.1cc_, D_1cc_ and D_2cc_ of the bladder and rectum. When we calculate the cumulative brachytherapy dose by summing up D_0.1cc_, D_1cc_ and D_2cc_ of the organs at risk for all the applications, we must bear in mind their inter-application displacement, as well as the fact that it is less likely that these volumes indeed received the calculated dose. That means that it is less likely that the worst case scenario shall indeed happen. Planning the brachytherapy of the inoperable cervical cancer on the basis of computer tomography is required for every application during the brachytherapy treatment.

## Figures and Tables

**TABLE 1.** Patient demographics

**Characteristics**	**Mean ± SD**
Age	52 ± 11
Cancer stage FIGO	
IIb	24 (80%)
IIIb	5 (16.7%)
IVa	1 (3.3%)

FIGO **=** International Federation of Gynecology and Obstetrics; SD = standard deviation

**TABLE 2.** The mean values, standard deviation, and minimum and maximum X, Y, and Z axis displacement of the brachytherapy dose received by the referential volumes of the bladder, and their absolute displacement / intensity of the vector (R) are shown for all applications in relation to the first one. The values are given in centimetres

		**Mean**	**SD**	**Minimum**	**Maximum**
^1^**X axis displacement**	**II-I applications**	−.29	1.78	−2.85	5.38
	**III-I applications**	.42	1.71	−2.68	4.26
	**IV-I applications**	−.04	2.11	−3.05	5.62
	**V-I applications**	−.07	2.45	−5.32	4.32
^2^**Yaxis displacement**	**II-I applications**	.29	.80	−1.73	1.93
	**III-I applications**	.29	.99	−1.62	2.43
	**IV-I applications**	.29	1.10	−2.00	2.45
	**V-I applications**	.20	1.28	−2.38	2.50
^3^**Zaxis displacement**	**II-I applications**	−.05	1.00	−2.13	1.48
	**III-I applications**	.34	1.35	−2.13	3.14
	**IV-I applications**	.20	1.42	−2.05	3.23
	**V-I applications**	−.09	1.52	−3.31	2.49
^4^**Vector magnitude (R)**	**II-I applications**	1.95	1.03	.68	5.52
	**III-I applications**	2.18	1.10	.72	4.88
	**IV-I applications**	2.53	1.09	.74	5.63
	**V-I applications**	2.83	1.32	.74	5.63

SD = standard deviation;

1 p = 0.59;

2 p = 0.98;

3 p = 0.54;

4 p = 0.02

**TABLE 3.** The mean values, standard deviation, and minimum and maximum X, Y, and Z axis displacement of the brachytherapy dose received by the referential volumes of the rectum, and their absolute displacement / intensity of the vector (R) are shown for all applications in relation to the first one. The values are given in centimetres

		**Mean**	**SD**	**Minimum**	**Maximum**
^1^**X axis displacement**	**II-I applications**	−.46	1.17	−2.82	1.51
	**III-I applications**	−.15	1.11	−2.32	2.56
	**IV-I applications**	−.39	1.67	−3.69	3.36
	**V-I applications**	−.44	2.07	−5.32	2.71
^2^**Yaxis displacement**	**II-I applications**	.24	1.01	−1.99	2.20
	**III-I applications**	.20	1.10	−3.13	2.81
	**IV-I applications**	.12	1.05	−2.71	1.69
	**V-I applications**	.37	1.48	−2.80	3.00
^3^**Zaxis displacement**	**II-I applications**	−.51	1.42	−3.68	2.41
	**III-I applications**	.18	1.50	−2.23	2.37
	**IV-I applications**	−.22	1.99	−4.55	3.40
	**V-I applications**	.52	1.55	−2.73	3.66
^4^**Vector magnitude(R)**	**II-I applications**	2.05	.77	.55	3.78
	**III-I applications**	2.01	.69	.23	3.74
	**IV-I applications**	2.48	1.31	.46	5.06
	**V-I applications**	2.78	1.22	.54	6.17

SD = standard deviation;

1 p = 0.87;

2 p = 0.87;

3 p = 0.08;

4 p = 0.012
